# Cortical Layer 1 and Layer 2/3 Astrocytes Exhibit Distinct Calcium Dynamics *In Vivo*

**DOI:** 10.1371/journal.pone.0002525

**Published:** 2008-06-25

**Authors:** Norio Takata, Hajime Hirase

**Affiliations:** Hirase Research Unit, Neuronal Circuit and Mechanisms Research Group, RIKEN Brain Science Institute, Wako, Japan; Temasek Life Sciences Laboratory, Singapore

## Abstract

Cumulative evidence supports bidirectional interactions between astrocytes and neurons, suggesting glial involvement of neuronal information processing in the brain. Cytosolic calcium (Ca^2+^) concentration is important for astrocytes as Ca^2+^ surges co-occur with gliotransmission and neurotransmitter reception. Cerebral cortex is organized in layers which are characterized by distinct cytoarchitecture. We asked if astrocyte-dominant layer 1 (L1) of the somatosensory cortex was different from layer 2/3 (L2/3) in spontaneous astrocytic Ca^2+^ activity and if it was influenced by background neural activity. Using a two-photon laser scanning microscope, we compared spontaneous Ca^2+^ activity of astrocytic somata and processes in L1 and L2/3 of anesthetized mature rat somatosensory cortex. We also assessed the contribution of background neural activity to the spontaneous astrocytic Ca^2+^ dynamics by investigating two distinct EEG states (“synchronized” vs. “de-synchronized” states). We found that astrocytes in L1 had nearly twice higher Ca^2+^ activity than L2/3. Furthermore, Ca^2+^ fluctuations of processes within an astrocyte were independent in L1 while those in L2/3 were synchronous. Pharmacological blockades of metabotropic receptors for glutamate, ATP, and acetylcholine, as well as suppression of action potentials did not have a significant effect on the spontaneous somatic Ca^2+^ activity. These results suggest that spontaneous astrocytic Ca^2+^ surges occurred in large part intrinsically, rather than neural activity-driven. Our findings propose a new functional segregation of layer 1 and 2/3 that is defined by autonomous astrocytic activity.

## Introduction

Astrocytes occupy a significant proportion of the cellular composition in the brain. Their functions have been considered to be logistic support for neurons, such as mediating energy metabolism for neurons or maintenance of extracellular medium concentrations [1], [2]. While the traditional roles of astrocytes have gained experimental support, evidence has accumulated to suggest bidirectional neuron-glia communications [3] and possible participation in neural information processing [4], [5]. For instance, astrocytes have been shown to release neuroactive molecules (“gliotransmitters”) such as glutamate, adenosine tri-phosphate (ATP), and acetylcholine (ACh), which could underlie astrocytic effects on neurons such as potentiated transmitter release and hetero-synaptic depression [6], [7]. At the signal reception end, astrocytes express a variety of neurotransmitter receptors including glutamate, purine, and ACh [8].

Although astrocytes lack fast sodium action potentials, astrocytic activity can be monitored by cytosolic calcium (Ca^2+^) level as neurotransmitters induce Ca^2+^ surge in astrocytes and many forms of gliotransmission are accompanied by cytosolic Ca^2+^ increase [9] (see also Fiacco et al. [10] for recent controversy). Two distinct forms of astrocytic Ca^2+^ surges are reported: (1) neural activity driven Ca^2+^ surges, and (2) neural activity independent “intrinsic” Ca^2+^ surges. The neural activity driven astrocytic Ca^2+^ surge is triggered by activation of metabotropic neurotransmitter receptors, which leads to production of inositol 1,4,5-triphosphate (IP_3_), and thereby to release of Ca^2+^ from the endoplasmic reticulum (ER) [3]. The mechanism of the “intrinsic” Ca^2+^ surge is not known, but it is also mediated by IP_3_ receptor of ER [11]. Although evidence suggesting a dynamic role of astrocytes through the Ca^2+^ activity is accumulating in *in vitro* experiments, an oft-expressed concern is the modification of cellular properties of astrocytes due to the invasive nature of the preparations [12].

Anatomically, neurons are organized in layers with characteristic cytoarchitecture in the intact cerebral cortex. Notably, with regard to cell bodies, L1 contains mostly astrocytes, while L2/3 is dominated with neuronal soma [13]. Physiologically, distinct network states such as the “synchronized” and “desynchronized” states emerges in different levels of vigilance [14] or anesthesia [15], which are reflected in field potential (EEG) recording. Previous *in vivo* imaging studies have reported astrocytic Ca^2+^ surges upon optimized sensory stimulation [16] or running behavior [17] and the existence of spontaneous astrocytic Ca^2+^ surges in juvenile rats (<P16) [18] and chronic seizures [19]. However, comparison of spontaneous activity of astrocytes in different layers of mature cerebral cortex or different brain states has not been addressed.

We asked whether spontaneous astrocytic Ca^2+^ dynamics (1) reflected cytoarchitectural differences of L1 and L2/3 and (2) changed during two distinct EEG states. We found spontaneous Ca^2+^ surges of astrocyte somata in cortical L1 were nearly twice more frequent than that of L2/3. Furthermore, Ca^2+^ dynamics of processes within an astrocyte were independent in L1 while those in L2/3 were synchronous. EEG state comparisons, as well as pharmacological experiments showed insensitivity of the spontaneous Ca^2+^ surges of astrocytes to neuronal activities, suggesting that the Ca^2+^ surges were in large part “intrinsic”, rather than neural activity driven. Our findings propose a new functional segregation of layer 1 and 2/3 that is defined by autonomous activity of astrocytes.

## Results

### Cytoarchitectural difference between layer 1 and layer 2/3

**Figure 1:**
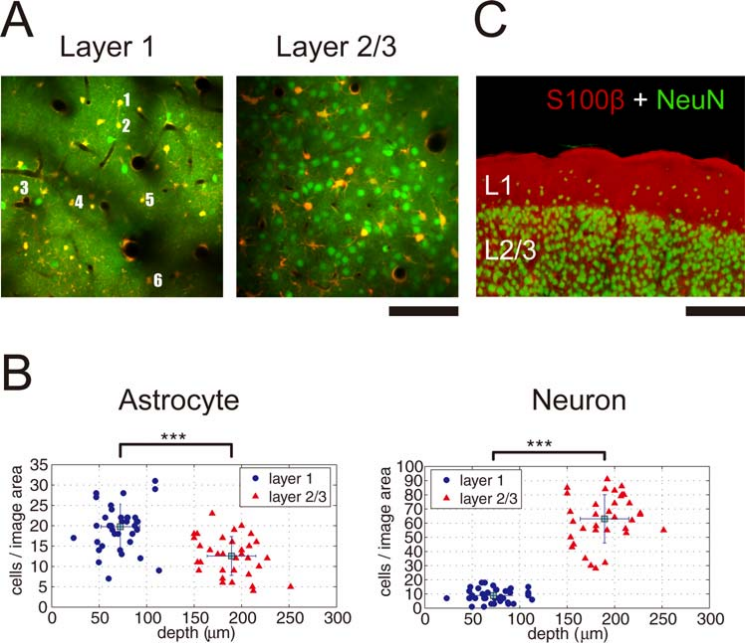
Distinct cytoarchitecture of cortical layer 1 and layer 2/3. A, Representative *in vivo* images of layer 1 (L1) and layer 2/3 (L2/3). The Ca^2+^ indicator Oregon Green 488 BAPTA-1 (OGB-1, green) stained both neurons and astrocytes while Sulforhodamine 101 (SR101, red) stained astrocytes. In L1, most of the cells were astrocytes, indicated by loading with OGB-1 and SR101 (red–yellow). In L2/3, neurons (SR101-negative) outnumber astrocytes (SR101-positive). Time course of Ca^2+^ transients of the numbered astrocytes (1–6) in L1 image is presented in Figure 2A (for L2/3, see Figure S1). Movies of Ca^2+^ transients for L1 and L2/3 are shown in Video S1 and S2. B, The packing densities of astrocytes and neurons are plotted against the depth from cortical pia. L1 (blue circle) and L2/3 (red triangle) were determined by the physical depth and appearance of astrocyte-neuron ratio. Counts of astrocyte and neuron were 19.8±5.6 and 8.9±4.5 in L1 (n = 38 imaging sites), 12.6±4.8 and 63.0±17.1 in L2/3 (n = 34) per imaged area (∼320×320 µm^2^) (mean±SD in all figures, otherwise noted). While L1 has more astrocytes than layer 2/3, neurons dominated in L2/3 (***p<0.001, Student's *t* test). C, Immunohistochemical staining for S100β (astrocyte marker, red) and NeuN (neuron marker, green) of a coronal cortical section. Glial dominance in L1 and neuronal dominance in L2/3 is evident. Scale bar: A, 100 µm; C, 200 µm.

Multi-cell bolus loading with the Ca^2+^ sensitive fluorescent dye Oregon Green 488 BAPTA-1 (OGB-1) and the astrocyte marker Sulforhodamine 101 (SR101) was performed in the primary somatosensory cortex of adult (>P28) male Sprague-Dawley rats. In agreement with previously published literature, both neurons and glial cells were loaded with OGB-1 and cells with astrocytic morphology were labeled with SR101 (Figure 1A) [20], [21]. The dye loaded area typically spread approximately 350 µm in diameter from the tip of the injection electrode and resulted in labeling in layer 1 (L1) and layer 2/3 (L2/3) of the somatosensory cortex (Figure 1A). The composition of labeled cells was substantially different between L1 and L2/3. In L1, the OGB-1 loaded cells predominantly co-localized with SR101 labeling, indicating that most of the loaded cells were astrocytes (Figure 1A left). In contrast, mixture of OGB-1-loaded neurons and astrocytes were observed in L2/3 (Figure 1A right). Counting of astrocyte and neuron cell bodies was performed in L1 (38 imaging sites) and L2/3 (34 imaging sites) from 18 animals. In L1, there were 19.8±5.6 astrocytes per imaging area (320 µm×320 µm≈0.1 mm^2^, estimated image place thickness = ∼2 µm) and L1 astrocytes were significantly more numerous than that of L2/3 (12.6±4.8, p<0.001, t-test, Figure 1B left). The number of SR101 negative cells, presumably neurons, was significantly higher in L2/3 than in L1 (L1: 8.9±4.5 vs. L2/3: 63.0±17.1, p<0.001, t-test, Figure 1B right). These differences reflect the cytoarchitectural difference of L1 and L2/3, as indicated by immunohistochemical staining of a fixed cortical slice against the astrocyte marker S100β and the neuron marker NeuN (Figure 1C).

### Single cell properties of astrocytic spontaneous Ca^2+^ surges

**Figure 2:**
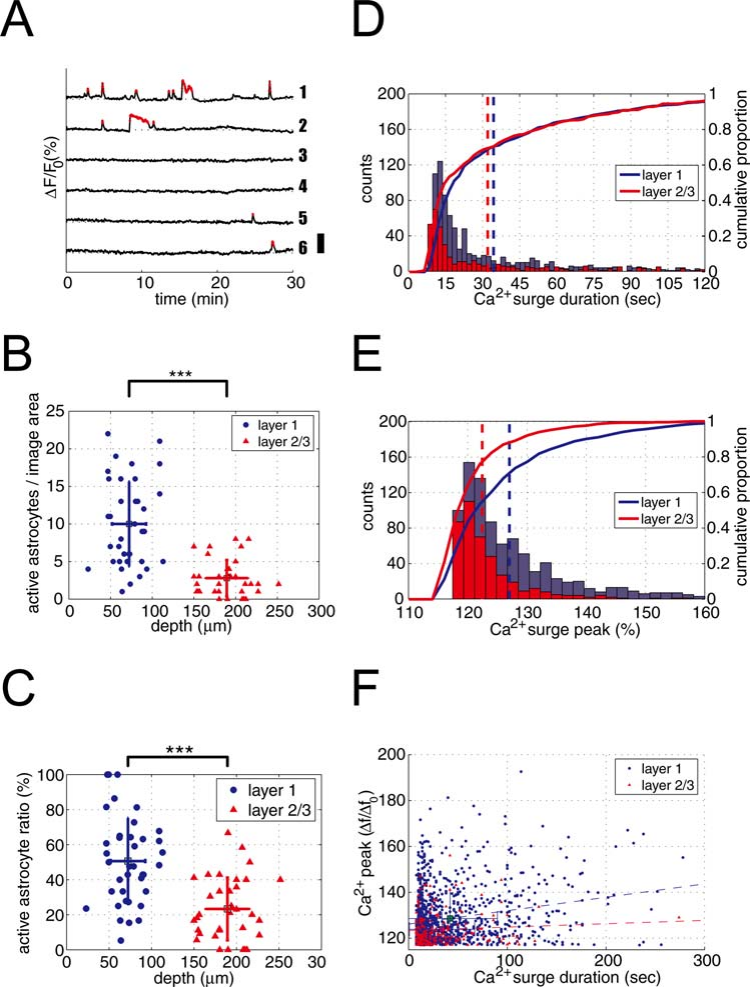
More astrocytes elicit spontaneous Ca^2+^ surges in layer 1 than in layer 2/3. A, Representative time course of spontaneous Ca^2+^ surges of astrocytes in L1. Numbers (1–6) correspond to astrocytes in Figure 1A. Each trace shows normalized fluorescence intensity of Ca^2+^ indicator OGB-1 from an individual astrocyte. Small red dots indicate period of Ca^2+^ surge. Vertical position of each trace was adjusted arbitrary to improve visibility. Scale bar: 50%. B, Number of astrocytes with at least one spontaneous Ca^2+^ surge (“active astrocyte”) per imaged area is plotted against the depth from the cortical pia. L1 had significantly more active astrocytes than L2/3. ***p<0.001, Student's *t* test. C, Proportion of active astrocyte for each experiment is plotted against the imaged depth. L1 had significantly higher percentage of active astrocytes than L2/3. ***p<0.001, Student's *t* test. D, Histograms (solid bars) and cumulative distribution (solid lines) of Ca^2+^ surge duration of astrocytes in L1 (blue) and L2/3 (red) are plotted. Vertical dotted lines indicate the mean duration of spontaneous Ca^2+^ surge. There was no significant difference in Ca^2+^ duration between L1 and L2/3. E, Histograms (solid bars) and cumulative distributions (solid lines) of Ca^2+^ surge peak of astrocytes in L1 (blue) and L2/3 (red) are plotted. L1 astrocytes had significantly higher spontaneous Ca^2+^ surge peak values. Vertical dotted lines indicate the mean Ca^2+^ surge peak (***p<0.001, Student's *t* test). F, Ca^2+^ surge peak values are plotted against Ca^2+^ surge duration in L1 (blue circle) and L2/3 (red triangle). There was a slight but significant correlation between Ca^2+^ peak value and duration in L1 (r≈0.24), but little correlation was observed in L2/3 (r≈0.07).

Imaging experiments (0.5 Hz, 30 min) totaled in 38 session for L1 and 34 sessions for L2/3 (see Video S1, S2 for representative movies). The total number of imaged astrocytes was 751 for L1 and 427 for L2/3. Figure 2A shows representative time-course of spontaneous Ca^2+^ surges of L1 astrocytes presented in Figure 1A (for L2/3 astrocytes, see Figure S1). We termed astrocytes which gave rise to at least one spontaneous Ca^2+^ surge as “active astrocyte”, and astrocyte without Ca^2+^ surge during the 30 min imaging session as “inactive astrocyte”. The total number of active astrocytes was 380 (50.6%) in L1 and 94 (22.2%) in L2/3. The average number of active astrocytes was 10.0±5.6 per imaging field in L1 and was significantly higher than 2.8±2.4 in L2/3 (p<0.001, t-test) (Figure 2B). In order to quantify the proportion of active astrocytes, we took the ratio of the number of active astrocytes and SR101 labeled cells for each experiment. The proportion of active astrocytes was also significantly higher in L1 than in L2/3 (50.6±24.6% vs. 23.3±17.9%, t-test, p<0.001) (Figure 2C). The mean number of spontaneous Ca^2+^ events per astrocytes (active and inactive) in thirty minutes was calculated to be 1.5±2.3 for L1 and 0.4±1.1 for L2/3 and the difference was significant (p<0.001, t-test). Among active astrocytes, L1 had 3.0±2.5 Ca^2+^ surges in thirty minutes and was significantly higher than L2/3 which had 2.0±1.7 Ca^2+^ surges (p<0.001, t-test). These astrocytic spontaneous Ca^2+^ events had of an average duration (see methods for definition) of 34.8±35.8s in L1 and 32.5±34.6s in L2/3 (Figure 2D). The durations of spontaneous astrocytic Ca^2+^ events resembled an exponential-like distribution. The mode and median of the Ca^2+^ event duration was 12∼14s and 22s for L1 and 10∼12s and 22s for L2/3, respectively. While the average duration of astrocytic Ca^2+^ surge in L1 and L2/3 did not differ significantly (p = 0.29, t-test, Figure 2D), the average peak value of the Ca^2+^ events' fluorescence intensities was significantly higher in L1 (127.0±0.4) than L2/3 (122.4±0.3) (p<0.001, t-test, Figure 2E). We questioned if there is a systematic relationship between the duration of a Ca^2+^ surge and the peak value of the Ca^2+^ events. Linear regression analysis revealed that there is small, yet significant correlation between the duration of the Ca^2+^ surge and the peak signal in L1 astrocytes (r≈0.24, p<0.001, Figure 2F). In contrast, there was not a significant correlation in L2/3 astrocytes (r≈0.07, p = 0.37, Figure 2F).

**Figure 3:**
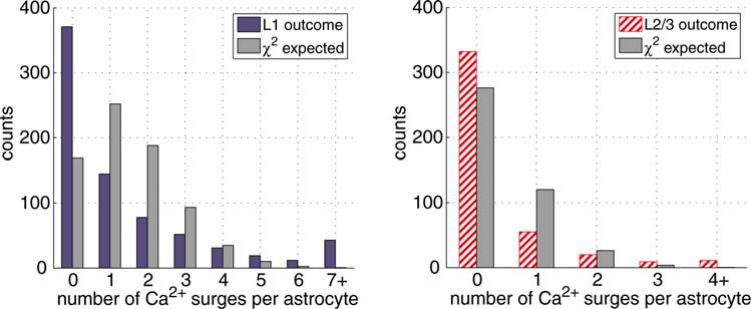
Ca^2+^ surges occurrence in astrocytes are non-uniform. The distribution of astrocytic spontaneous Ca^2+^ surge frequency in L1 (blue bar, left graph) or L2/3 (red hashed bar, right graph) was compared with the Poisson distribution (grey bar) that represents unbiased occurrence probability of Ca^2+^ surges among astrocytes. Distribution of experimental data deviated significantly from the Poisson distribution (L1: χ^2^ = 4586, d.f. = 5, p<0.001; L2/3: χ^2^ = 1316, d.f. = 2, p<0.001). “7+” and “4+” indicate summed counts of Ca^2+^ surge-number equal to or larger than 7 and 4, respectively. Note the large deviation between Poisson distribution and experimental results, especially at Ca^2+^ surge counts 0∼1 and 7+ (L1) or 4+ (L2/3).

To test if there is a subpopulation of astrocytes that are more likely to elicit spontaneous Ca^2+^ surges, we calculated the histogram representing the frequency of Ca^2+^ surges of an astrocyte per thirty minutes for each layer (Figure 3 ‘L1 outcome’ and ‘L2/3 outcome’). The histogram was compared with the Poisson distribution (Figure 3 ‘χ^2^ expected’). The Poisson distribution reflects unbiased random occurrences of Ca^2+^ surges using the mean counts of astrocytic Ca^2+^ events measured in the experiments (1.5 and 0.4 Ca^2+^ events/cell/30 min in L1 and L2/3, respectively). The experimental distribution deviated significantly from the Poisson distribution (L1, χ^2 = ^4586, d.f. = 5, p<0.001; L2/3, χ^2 = ^1316, d.f. = 2, p<0.001, Figure 3). The result suggests that the occurrence of Ca^2+^ surges in astrocytes is not a Poisson process and hints the existence of a subpopulation of active astrocytes.

**Figure 4:**
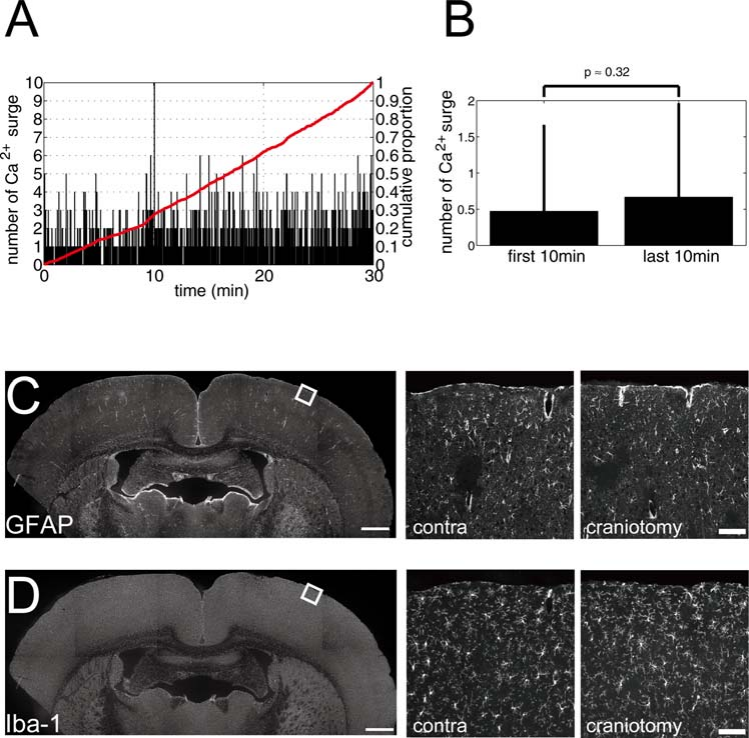
Condition of craniotomy assessed with spontaneous Ca^2+^ surge occurrence and immunohistochemistry. A, Number of spontaneous Ca^2+^ surges of astrocytes of all experimental data was plotted against imaged time. Red line indicates the cumulative proportion. Apparently uniform occurrence probability of spontaneous Ca^2+^ surge was observed. B, Comparison of number of Ca^2+^ surges during the first and the last 10 min of the imaging period. No significant difference was observed (p = 0.32). C, Wide field view of immunostained cortex with astrocyte specific antibody, GFAP (left). Square indicates the imaged area with craniotomy, which is displayed in an expanded view (right, ‘craniotomy’) and compared with the contralateral side of the corresponding cortical area (middle, ‘contra’). Comparable shape of astrocyte was acknowledged. D, Wide field view of immunostained cortex with microglia specific antibody, Iba-1 (left). Square indicates the imaged area with craniotomy, which is displayed in an expanded view (right, ‘craniotomy’) and compared with the contralateral side of the corresponding cortical area (middle, ‘contra’). Similar morphology of microglia was observed. Scale bar: C, D, 1 mm (left); 100 µm (middle and right).

As the layer difference in the proportion of active astrocytes may be due to photodamage by laser irradiation [22], we plotted the time course of the occurrences of astrocytic Ca^2+^ surges, showing uniform probability of Ca^2+^ surge occurrence during the imaging session (Figure 4A). We also compared the average number of spontaneous Ca^2+^ surges per astrocyte in the first and the last 10 min of the 30 min imaging session, revealing no significant difference (0.5±1.2 vs. 0.7±1.3, t-test, p≈0.32, Figure 4B). Another factor which may influence Ca^2+^ activity of astrocytes is the surface exposure due to craniotomy [23]. To asses the effect of craniotomy, we compared morphology of astrocytes and microglia by immunohistochemistry using GFAP (glial fibrillary acidic protein; astrocyte marker) and Iba-1 (ionized calcium-binding adaptor molecule 1; microglia marker) antibodies after imaging experiments, showing intact morphology of these glia (Figure 4C, D). To further assess the effect of craniotomy or laser irradiation, we have computed the correlation between the imaged depth and the proportion of spontaneously active astrocytes within each of the layers (see Figure 2C). Neither in L1 (r = −0.09, p = 0.58) or L2/3 (r = 0.11, p = 0.53), there was no significant correlation between the depth of imaging and the proportion of active astrocytes within each layer. These results suggest that the difference in the proportion of active astrocytes between L1 and L2/3 was unlikely to be due to artifacts of craniotomy or laser photodamage.

### Spatio-temporal dynamics of astrocytic Ca^2+^ surges

**Figure 5:**
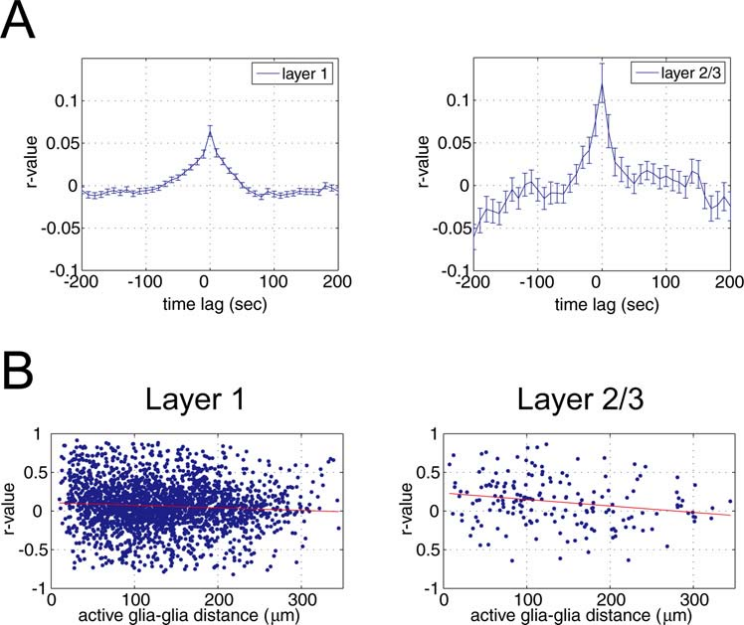
Spatio-temporal correlation of Ca^2+^ activity of active astrocytes was low. A, Mean cross-correlation of Ca^2+^ sensitive fluorescence intensity (ΔF/F_0_) of pairs of “active astrocytes” (astrocyte with as least one Ca^2+^ surge) in L1 (2300 pairs of active astrocytes) and L2/3 (181 pairs). Correlation coefficients (r-value) for both L1 and L2/3 suggest that temporal correlation among active astrocytes is weak (r<0.15). B, Relationship between distance of active astrocytes and the magnitude of correlation at zero time lag. The correlation coefficients were −0.07 and −0.21 for L1 and L2/3, respectively, showing little association between cell to cell distance and synchrony of Ca^2+^ surges when computed over the whole population.

To investigate temporal characteristics of Ca^2+^ surges among astrocytes, pair-wise cross-correlograms of OGB-1 intensity (ΔF/F_0_) were calculated (Figure 5A, n = 2300 and 181 pairs of active astrocytes in L1 and L2/3, respectively). The correlation coefficient (r-value) had less than 0.15, indicating little temporal correlation among Ca^2+^ surges of astrocytes at the population level. For the analysis of spatial correlation of astrocytic Ca^2+^ surge, the magnitude of the zero time lag correlation coefficient was plotted against distance between pairs of active astrocytes (Figure 5B). Little relationship was observed between these variables (r = −0.07 and −0.21 for L1 and L2/3, respectively). These data suggest that, on average, Ca^2+^ surges of astrocytes have little spatial and temporal correlation.

**Figure 6:**
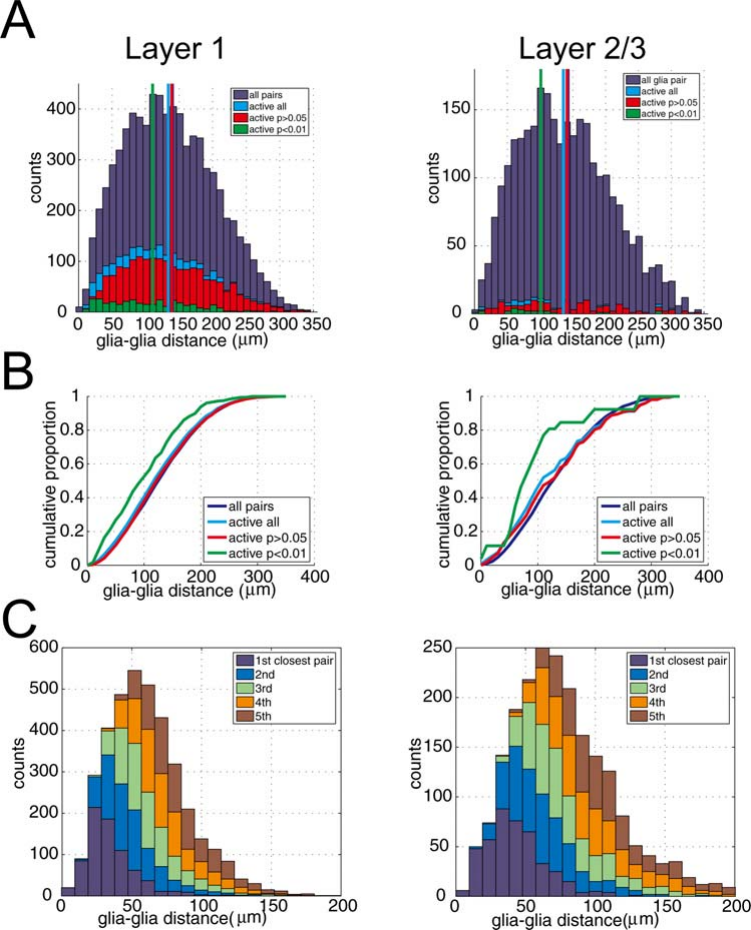
Pairs of synchronously active astrocytes were located closer. A, Histograms of cell to cell distance of astrocyte pairs. “All pairs” contains pairs of astrocytes whether they have Ca^2+^ surge or not during the 30 min imaging session (L1: 7625 pairs, L2/3: 2843 pairs). “Active all” includes pairs of an astrocyte which has at least one Ca^2+^ surge (L1: 2481 pairs, L2/3: 181 pairs). Pairs of “active astrocyte” were separated into two categories: “active p>0.05” and “active p<0.01” according to their synchrony of Ca^2+^ surges. Synchrony of Ca^2+^ surge between a pair of astrocytes was estimated with Fisher's exact test (see method). Highly synchronized “active p<0.01” pairs are in closer proximity than other pairs of astrocytes (p<0.001 in L1, p<0.02 in L2/3. ANOVA followed by Tukey's HSD test). Vertical dotted lines indicate the mean distance of astrocyte pairs. Cumulative plot for the distributions are plotted in B. C, Histograms of distance of all the imaged astrocytes separated in the order of physical proximity. “1st closest pair” indicates the distribution of distances of a pair of closest astrocytes. The histogram shows that pairs of astrocyte with synchronized Ca^2+^ surge (“active p<0.01”) are not necessarily the closest pairs.

Although there was little spatial and temporal correlation among active astrocytes (Figure 5A, B), we sporadically observed highly correlated Ca^2+^ surges among astrocytes (see Figure 2A). To analyze the spatial relationship of those apparently correlated astrocytes, we segregated pairs of active astrocytes that are significantly synchronized by means of the Fisher's exact test (see Methods). Out of 2662 (L1: 2481, L2/3: 181) pairs of active astrocytes, 382 (L1: 356, L2/3: 26) pairs were found to be significantly synchronized (p<0.01), and 2038 (L1: 1886, L2/3: 152) pairs had p values larger than 0.05 in the Fisher's exact test which we defined as the non-synchronous pairs. First, we compared the distribution of cell to cell distance between all imaged astrocytes (7625 pairs in L1 and 2843 pairs in L2/3, denoted as ”all pairs” in Figure 6A) and only active astrocytes (2481 pairs in L1, and 181 pairs in L2/3, denoted as “active all” in Figure 6A). The mean distance of “active all” pairs was marginally smaller than that of “all pairs” (average distance of “active all”: 134.4±67.8 µm vs. “all pairs”: 140.7±67.8 µm. t-test, p<0.001). Next, pairs of active astrocytes were separated as synchronized pairs (“active p<0.01”) and non-synchronized pairs (“active p>0.05”). We found the mean distance among the synchronized astrocytes to be significantly smaller than that of the non-synchronized pairs of astrocytes (L1: 109.7±63.2 µm vs. 139.1±67.5 µm, t-test p<0.001. L2/3: 101.1±70.5 µm vs. 141.2±79.0 µm, p<0.02) (Figure 6A). The mean distance among the non-synchronized pairs was similar to that among whole astrocytes (“all pairs”) (L1: 139.1±67.5 µm vs. 140.7±67.8 µm, t-test, p = 0.32. L2/3: 141.2±79.0 µm vs. 142.8±69.9 µm, t-test, p = 0.79 in L2/3), suggesting synchronous Ca^2+^ surge occurs between closely located astrocytes. Cumulative plot of glia-glia distance shows distinct mean distances among pairs of synchronized and non-synchronized astrocytes (Figure 6B). Next, we computed distances between pairs of 1st, 2nd, 3rd, 4th, and 5th closest astrocytes (Table 1, Figure 6C). In L1, the average distance between synchronized pairs of astrocytes (109.7±63.2 µm) was larger than that of the 5th closest pairs (89.5±27.5 µm). In L2/3, the average distance between synchronized pairs of astrocytes (101.1±70.5 µm) corresponds to the 4th closest pairs (100.5±34.4 µm). These results indicate that synchronized astrocytes are closer pairs than average, but not necessarily the closest pairs.

**Table 1:** Distances between pairs of astrocytes in closest order.

	1st	2nd	3rd	4th	5th
Layer 1	36.3±18.5	52.5±21.4	65.6±23.2	77.8±24.9	89.5±27.5
Layer 2/3	44.1±22.2	65.1±26.1	82.3±31.1	100.5±34.4	115.1±39.2

Numbers represent mean±SD (µm).

### Spontaneous Ca^2+^ surge of astrocytes occurred independent on neural activity

**Figure 7:**
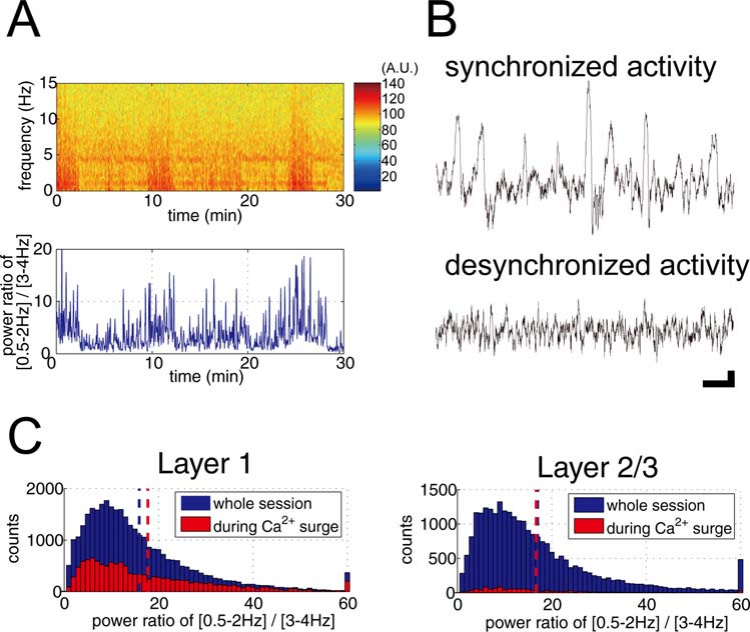
Low corelation between cortical EEG states and spontaneous astrocytic Ca^2+^ surges. A, Representative EEG spectrogram of cortex shows alternating epochs of synchronized and desynchronized neuronal activity under urethane anesthesia (upper graph). Synchronized and desynchronized activities are characterized by high EEG power of slow wave (0.5–2 Hz) and theta range frequency (3–4 Hz), respectively. To quantify states of EEG activity, power ratio of EEG frequency of 0.5–2 Hz over 3–4 Hz was calculated (lower graph). B, Representative EEG traces for synchronized and desynchronized states. Synchronized activity is characterized by high amplitude slow wave. Desynchronized state of EEG is characterized as smaller and rapid activity. Scale: 1 sec (horizontal), 100 µV (vertical). C, Histogram of EEG power ratio of [0.5–2 Hz] and [3–4 Hz] during whole imaging session (blue bar) and during periods with astrocytic spontaneous Ca^2+^ activity (red bar). Vertical dotted lines indicate the mean ratio. Mean ratio during whole imaging session and periods with astrocytic Ca^2+^ surges did not differ significantly (Wilcoxon rank sum test, p = 0.35 for L1, p = 76 for L2/3).

**Figure 8:**
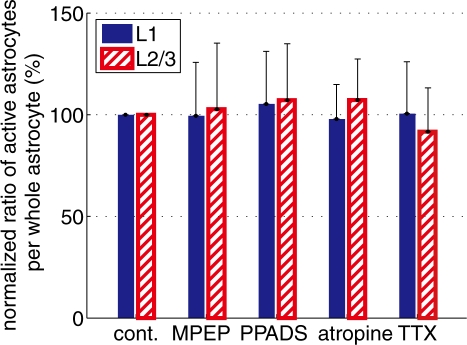
Spontaneous Ca^2+^ surges of astrocytes were not affected by pharmacological inhibitors. Percentage of active astrocytes in inhibitor experiments was compared with control experiments. The mGluR5 blocker MPEP, or the P2Y receptor blocker PPADS, the voltage gated sodium channel blocker TTX, or the muscarinic acetylcholine receptor blocker atropine had no effects on the occurrences of spontaneous astrocytic Ca^2+^ surges.

The local field potential (EEG) and multi-unit activity was measured in the vicinity of the imaged area. The local field potential elicited alternating epochs of synchronized activity and desynchronized activity under urethane anesthesia (Figure 7A upper panel). In the synchronized state, discharge activity of neurons oscillates synchronously with a slow (0.5–2 Hz) rhythm (Figure 7B upper trace) [15], [24]. In the desynchronized state, the amplitude of the local field potential fluctuation falls down to a few hundred microvolt range and faster oscillations appear (Figure 7B lower trace). In order to determine if the spontaneous astrocytic glial activity depends on the EEG states, we have compared ratio of EEG power (synchronized state [0.5–2 Hz]/desynchronized state [3–4 Hz]) during the whole experimental session and during periods with astrocytic Ca^2+^ surges (Figure 7A lower panel). In L1, the mean EEG power ratio during whole experimental session and during astrocytic Ca^2+^ surge were 15.5±6.7 and 14.4±7.6, respectively (Wilcoxon rank sum test, p = 0.35) (Figure 7C left). In L2/3, the mean EEG power ratio was 16.3±9.1 and 17.5±11.2, respectively (Wilcoxon rank sum test, p = 0.76) (Figure 7C right). These data show that the EEG power ratio did not differ significantly between periods of Ca^2+^ surge and Ca^2+^ quiescence, implying spontaneous neuronal activity did not have significant effects on Ca^2+^ activities of astrocytes. To analyze the effect of action potential dependent release of neurotransmitters on astrocytic Ca^2+^ activity, tetrodotoxin (TTX, 2 µM) was applied on the cortical surface for >30 min before Ca^2+^ imaging. TTX had no noticeable effect on the ratio of astrocytes with spontaneous Ca^2+^ surges (Figure 8, L1: 100±26%, n = 12 imaging sites; L2/3: 91±22%, n = 12), while TTX significantly reduced percentage of active neurons in L2/3 from 12±11% to 1±4% during the 30 min imaging session (t-test, p = 0.002), suggesting no significant effect of TTX on spontaneous Ca^2+^ surges of astrocytes in L1 and L2/3.

### Astrocytic spontaneous Ca^2+^ surge is not mediated by glutamate-, purinergic-, or acetylcholine receptors

To assess the possibility that Ca^2+^ surges of astrocytes are due to action potential independent release of neurotransmitter or gliotransmitter such as glutamate and ATP, pharmacological experiments with antagonists were performed. As a result, we found neither MPEP (mGluR5 antagonist, systemic injection 10 mg/kg), or PPADS (P2 purinergic receptor antagonist, surface application, 1 mM) had no noticeable effect on percentage of astrocytes with spontaneous Ca^2+^ surges (Figure 8, MPEP: in L1, 99±26%, n = 14 imaging sites; in L2/3, 102±33%, n = 12. PPADS: in L1, 105±26%, n = 6; in L2/3, 107±28%, n = 6). Because L1 contains distinctively higher density of acetylcholine axons than L2/3 [25], effect of atropine (mAChR antagonist, surface application on the pia, 1 mM) was also investigated. No significant effect on the ratio of active astrocytes, however, was observed (Figure 8, L1: 97±17%, n = 12; L2/3: 107±20%, n = 12). Although those inhibitors (MPEP, PPADS, and atropine) did not have significant influences on astrocytic Ca^2+^ surges, effectiveness of the inhibitors on the somatosensory cortex was confirmed by measuring EEG or ATP-induced astrocytic Ca^2+^ response (Figure S2) [26]–[28]. These results suggest that spontaneous Ca^2+^ surges of astrocytes occur independently on metabotropic glutamate receptors, purinergic receptors, or cholinergic receptors.

### Processes of astrocyte in layer 1 and layer 2/3 operated differently

**Figure 9:**
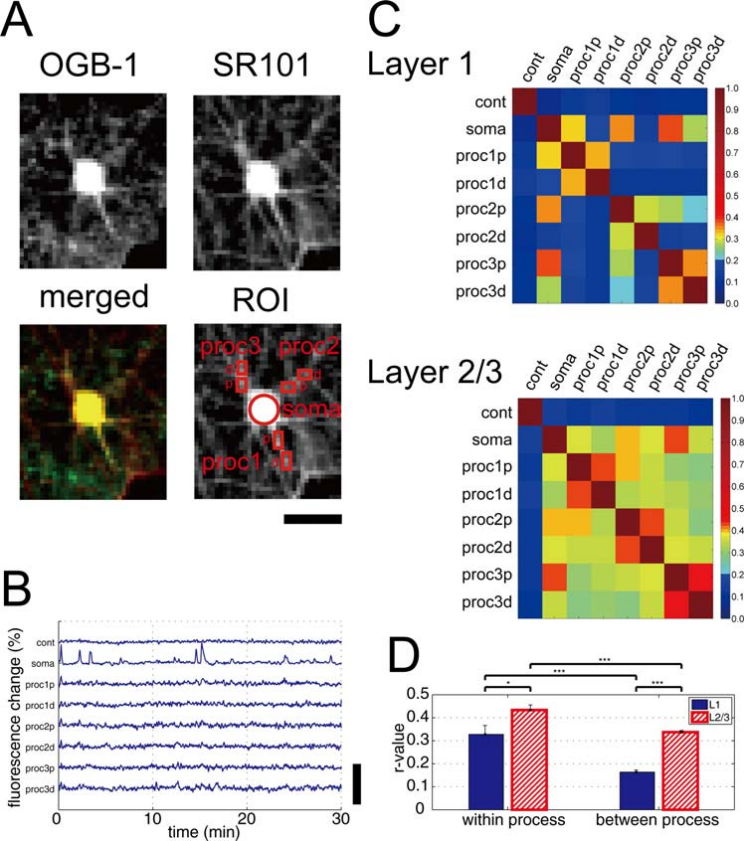
Dynamics of Ca^2+^ activities of astrocytic processes differ between layer 1 and layer 2/3. Astrocyte's soma and processes were identified with astrocyte specific SR101 image (A). Three primary processes (proc) of an astrocyte were arbitrarily selected and two rectangular ROIs were placed at each primary process. One ROI was placed at proximal (p) part of the process and the other was at distal (d) part. Ca^2+^ activity was monitored throughout the imaged session for each of the chosen region (B). As a control, one ROI was placed at neuropil at least 100 µm away from the astrocyte. The control ROI had the same size as the ROI of astrocytic soma. Representative image (A) and Ca^2+^ traces (B) are shown for an astrocyte in L1. For an astrocyte in L2/3, see Figure S3. C, Pearson's linear correlations (r-value) between Ca^2+^ traces of ROIs were calculated for L1 (30 processes of 10 astrocytes from 3 animals, upper panel) and L2/3 (30 processes of 10 astrocytes from 3 animals, lower panel). The correlation coefficient of ‘within-process’ (e.g. proc1p vs. proc1d) ROIs was high in both L1 & L2/3. Note that the between-processes correlation was high only in L2/3 (e.g. proc1p vs. proc2p in layer 2/3). D, Comparisons of the average correlation coefficient of within-process and between-process for L1 and L2/3. Note that while the correlation coefficient of ‘within process’ is high (r>0.3) in both L1 and L2/3, the correlation of ‘between process’ is high only in L2/3. *p<0.05, ***p<0.001; one-way ANOVA followed by Tukey's HSD test. Scale bar: A, 20 µm; B, 100%.

With our imaging configuration of one pixel for ∼1 µm^2^, primary processes of astrocytes could be detected with SR101 labeled image. Three clearly distinguishable primary branches were chosen for each astrocyte and two regions of interest (ROIs) were placed at each of the branches, one being more proximal (p), the other more distal (d) to the soma (Figure 9A). At astrocytic processes, fluctuation of the fluorescent Ca^2+^ indicator was generally smaller and the noise level of ΔF/F_0_ signal was relatively large when compared to somatic signal (Figure 9B). Therefore, we assessed Ca^2+^ dynamics at astrocytic processes by taking the pair-wise Pearson product-moment correlation coefficient (r-value). The correlation coefficient was calculated for pairs of ROIs (Figure 9C). Both in L1 and L2/3, ROIs within the same process of astrocyte had weak but positive association (e.g. proc1p vs. proc1d in L1 had r∼0.34, proc2p vs. proc2d in L2/3 had r∼0.43), indicating that Ca^2+^ activity of within-processes was correlated. On the other hand, while ROIs placed at different processes had little association in L1 (e.g. proc1p vs. proc2p had r∼0.19), in the case of L2/3, Ca^2+^ traces of ROIs placed at different processes of astrocyte had positive association (e.g. proc1p vs. proc2p had r∼0.39). The average correlation coefficients of within-process and between-process in L1 and L2/3 were summarized in Figure 9D. While the correlation of ‘within-process’ was high (r>0.3) in both L1 and L2/3 (L1: 0.33±0.21, n = 30 processes; L2/3: 0.43±0.12, n = 30 processes), the correlation of ‘between-process’ was high only in L2/3 (L1: 0.16±0.14, n = 240 processes; L2/3: 0.34±0.11, n = 240 processes). These data suggest that processes of astrocyte in L1 operated independently, while those in L2/3 were synchronous.

## Discussion

We investigated spontaneously occurring astrocytic calcium (Ca^2+^) surges in layer 1 (L1) and layer 2/3 (L2/3) of the somatosensory cortex of adult (>P25) rats *in vivo* in the absence of sensory input. Continuous imaging of the cortical areas for thirty minutes revealed long lasting (>10 s) spontaneous astrocytic Ca^2+^ surges with a frequency below one per ten minutes. We found there were twice as many “active astrocytes” (astrocytes with at least one Ca^2+^ surge) in L1 than L2/3. The spontaneous Ca^2+^ surges under our experimental condition were not influenced by the background neural activity and appeared to be originated intrinsically. Furthermore, process-level Ca^2+^ dynamics among different branches arising from an astrocyte appeared to be independent in L1, while those in L2/3 had significant synchrony. These results suggest that astrocytes in L1 and L2/3 have distinct modes of activity depending on their surrounding cytoarchitecture. These findings are discussed in detail below.

### Spontaneous somatic Ca^2+^ surges of single astrocytes

At the somatic level, we found there was generally higher astrocytic Ca^2+^ activity in L1 than L2/3. For instance, astrocytes that elicit spontaneous Ca^2+^ surges were more numerous in L1 than L2/3 (Figure 2B). However, this result may be intuitive, as the packing density of astrocytes in L1 is higher than that in L2/3 (Figure 1B). When the proportion of the spontaneously active astrocytes was computed, it became apparent that L1 astrocytes were nearly twice as likely to give rise to Ca^2+^ surges as that of L2/3 (Figure 2C). Furthermore, the average frequency of Ca^2+^ surges in active astrocytes was higher in L1 than L2/3. The mean magnitude of a Ca^2+^ surge was also significantly greater in L1 than L2/3, though the averaged duration was similar (Figure 2D, E).

One potential interpretation of the layer differences of astrocytic calcium dynamics is that the preparation of cranial window had nociceptive effects on astrocytes in the superficial L1 which resulted in turning the astrocytes to be ‘reactive’, which is indicated by increased expression of glial fibrillary acidic protein (GFAP) [23]. Accordingly, craniotomy might have modified the astrocytic Ca^2+^ dynamics. Our post-mortem immunohistochemical analysis, however, showed comparable expression of GFAP on both the imaged hemisphere and the contralateral control side, indicating that the astrocytes had not become reactive (Figure 4C). We have also examined the morphological characteristics of microglia, which has many ramified branches projected from soma in their normal condition. When the tissue is damaged, microglia has abnormal appearance, having amoeboid shapes having no or few processes or extending most of their branches toward the pial surface [23]. We observed similar morphology of microglia between the imaged hemisphere and the contralateral control side, indicating healthy microglial status in the imaged area (Figure 4D). These results suggest intact cortex at the imaged area in our experiments although nociceptive effects of craniotomy in chronic conditions have been reported previously [23]. This discrepancy about effects of craniotomy on the cortex may be due to the size of craniotomy. The previous study using adult mice demonstrated the nociceptive effects such as activated astrocytes and microglia by large craniotomy (5×5 mm^2^) [23], that exceeded the average length of bregma to lambda distance of a mouse (4.21 mm) [29]. In our experiments using adult rats, the size of craniotomy (≤1.77 mm^2^) was more than ten fold smaller. In fact, no nociceptive effects were reported using adult mice when small craniotomy was made [30].

It is notable that reactive astrocytes have been reported to display attenuated spontaneous Ca^2+^ activity [31]. In our experiments, astrocytes in L1, which would be more vulnerable to craniotomy-induced nociceptive stimulus, had more spontaneous Ca^2+^ surges than L2/3 (Figure 2C). Next, if the preparation of a cranial window has any effect on Ca^2+^ activity of an astrocyte, the activity level of spontaneous Ca^2+^ surges should depend on the physical depth of imaging within L1 or L2/3. No significant correlation, however, was observed between the spontaneous activity level and the physical depth within a cytoarchitecturally defined layer (Figure 2C. see result). While the influence of cranial window preparation may not be excluded completely, the careful surgical procedure minimized the artificial modification of the imaged area in our experimental condition.

Another interpretation of the layer difference is the effect of photostimulation to astrocytes by laser irradiation. It has been reported that imaging with excessive laser power artificially induced Ca^2+^ surges in astrocytes within a few minutes of laser irradiation [16], [22]. Such possibility is unlikely in our experiments because occurrence probability of Ca^2+^ surges of astrocyte appeared unbiased during the thirty minutes of imaging session (Figure 4A). Furthermore, the number of astrocytic Ca^2+^ surges in the first and last 10 min of imaging session was statistically indistinguishable, showing little effect of laser irradiation (Figure 4B). In addition, the dwell time of laser was minimum available (2 µs/pixel) in our experiments. As another possibility, temperature difference between cortical L1 and L2/3 during imaging experiments might cause cellular Ca^2+^ surge [32], [33], but we minimized laser power and kept a physiological brain temperature by perfusing warm solution. Taken together, we concluded that the layer difference of astrocytic Ca^2+^ activity was not due to artificial effects of craniotomy preparation or photostimulation.

An alternative physiological interpretation is that the layer difference of astrocytic Ca^2+^ dynamics reflects cytoarchitectural differences between L1 and L2/3. For instance, in L1 of the cortex, the cellular population is predominated by the presence of astrocytes. There is a sparse population of GABAergic interneurons, and gultamatergic cells are not generally present. In contrast, neurons are the major cell types in L2/3, and the mean astrocyte-to-neuron ratio was 1∶5 in our experiments (Figure 1B), which was in good agreement with previous report [34]. We computed that there was 1.6 times higher density of astrocytes in L1 than in L2/3. These cytoarchitectural differences led us to postulate that layer dependent neural activities may give rise to the differential astrocytic Ca^2+^ dynamics.

### Origin of spontaneous astrocytic Ca^2+^ activity

Under urethane anesthesia, the cortical field potential is characterized by the appearance of alternating epochs of large amplitude slow oscillations and desynchronized activities (Figure 7A, B). We fond that the EEG power ratio did not differ significantly between the different EEG states, implying that spontaneous astrocytic Ca^2+^ surges occur independently of EEG states. To further investigate the independence of spontaneous astrocytic Ca^2+^ surge on neuronal activity, neuronal action potential was suppressed by bath application of TTX. While TTX reduced Ca^2+^ increase of neurons, it did not change the ratio of active astrocytes (Figure 8), suggesting that spontaneous Ca^2+^ surge of astrocytes was independent of action potential triggered release of neurotransmitter. As L1 of somatosensory cortex is distinguished by receiving approximately sixty percent denser cholinergic innervation from the basal forebrain than L2/3 [25], we tested the significance of cholinergic input by blocking mAChRs with atropine. Inhibition of mAChRs, however, did not have a significant effect on the ratio of active astrocytes (Figure 8). These results suggest that spill over of neurotransmitters is too low to induce Ca^2+^ surges in astrocytes during the anesthetized state without peripheral stimulation. Entry of extracellular free Ca^2+^ through voltage gated Ca^2+^ channel on astrocytes is also unlikely because astrocytic membrane potential *in vivo* is retained below −75 mV under urethane anesthesia as we reported recently using *in vivo* intracellular recording method [35]–[37].

Gliotransmission of glutamate or ATP from astrocytes [38] and spontaneous neurotransmitter release in the absence of action potentials [39] are well established using *in vitro* preparations. These action potential independent mechanisms of transmitter release may activate mGluRs and P2YRs to give rise to astrocytic Ca^2+^ surges *in vivo*. In order to test such possibilities, we investigated effects of blocking mGluR5s by MPEP and P2YRs by PPADS. Our results show that neither MPEP nor PPADS had a significant effect on spontaneous Ca^2+^ surges (Figure 8), which is in contrast with Ca^2+^ surges evoked by sensory stimulation [16] or in epileptic conditions [19]. These results suggest that the spontaneous Ca^2+^ surges have little causal relationship with the on-going neuronal activity and that gliotransmitters such as glutamate or ATP are not involved in the somatic spontaneous Ca^2+^ surges. Taken together, the initiation of a spontaneous somatic Ca^2+^ surge is likely to be of intrinsic origin, rather than neural activity driven. Previously, Nett et al. has reported intrinsic Ca^2+^ surges of astrocytes in hippocampal slices prepared from juvenile mice [11]. They showed insensitivity of intrinsic Ca^2+^ surges of astrocytes to inhibitors such as MPEP, PPADS, and TTX. Our *in vivo* observation of spontaneous Ca^2+^ surges of astrocytes favors the idea of “intrinsic” Ca^2+^ surge of astrocytes.

### Spatiotemporal dynamics of somatic Ca^2+^ surges of astrocytes

We found that temporal synchrony of spontaneous Ca^2+^ surges among astrocytes was generally weak (Figure 5A) and there was no significant correlation between cell-to-cell distance and degree of synchrony between a pair of astrocytes (Figure 5B). These results on adult cortex are in good accordance with our previous results using juvenile rats [18]. Despite the overall appearance of low correlation, apparent synchronous Ca^2+^ surge was observed occasionally (Figure 2A). In fact, our analysis showed that pairs of astrocytes which showed synchronous Ca^2+^ surge were located more proximally to each other than the other pairs such as astrocytes without Ca^2+^ surge or astrocytes with a-synchronous Ca^2+^ increase (Figure 6A, B). However, it is noted that distance between pairs of these synchronized astrocytes was not necessarily the nearest pairs of astrocyte (Figure 6C). Considering EEG states and neural activity did not have significant effect on Ca^2+^ surges of astrocytes (Figure 7 and 8), the synchronized Ca^2+^ surge of astrocytes was not likely to be driven by spillover of neurotransmitters. Engagement of gliotransmission on the synchronized Ca^2+^ surge is also unlikely because pharmacological experiments showed that antagonist against mGluR and P2Y did not change the ratio of active astrocytes (Figure 8). Possible mechanism for the synchronous Ca^2+^ surge would be direct contact of astrocytic pairs through gap junctions at their peripheral processes. Although astrocytic soma has diameter of only ∼10 µm, the average distance of pairs of synchronous astrocytes (L1: 109.7±63.2 µm, L2: 101.1±70.5 µm) could be achieved because an astrocyte has been shown to have processes more than 60 µm occasionally [40]. Another possibility is that the somatic spontaneous Ca^2+^ surges are evoked by ligands that are secreted by a more diffuse secretory system like circulation and astrocytes that express sufficient amount of corresponding receptors.

### Ca^2+^ surges of astrocytic processes

We found that Ca^2+^ dynamics in processes of an astrocyte in L1 were independent, while that in L2/3 showed higher degree of synchrony (Figure 9C). Because astrocytic processes have direct contact with synapses [41] and astrocytes are hypothesized to work for information routing [5], it is intriguing to postulate that the difference of modes of astrocytic Ca^2+^ activity between L1 and L2/3 reflects signals which astrocytic processes handle. Indeed, while L2/3 of the somatosensory cortex receives input from layer 4 which conveys signals from the thalamus whose afferent inputs have been shown to be synchronous [42], L1 is the principal target of higher order cortical areas [43]–[45]. It is further tempting to consider afferents from the higher order processing that have a different spatial arrangement from primary sensory information that comes from thalamus and that astrocyte process level Ca^2+^ fluctuation reflects the input characteristics to the local circuit. Further experiments that focus on Ca^2+^ activity of astrocytic processes are necessary to reveal relationship between neuronal and astrocytic activities.

## Materials and Methods

### Subjects and surgery

Mature male Sprague-Dawley rats (postnatal 4–6 weeks old) were used for this study. Animals were deeply anesthetized with 1.7 g/kg urethane and the body temperature was maintained at 37.5 degrees with a regulated heating pad (TR-200, FST, CA, USA) throughout the surgery and following imaging experiment. Physiological saline containing dextrose (5% w/v) was injected subcutaneously as necessary (up to 10 ml/kg/hr) to maintain fluid balance. After the skull was exposed, a metal frame was attached to the skull using a dental acrylic (Fuji LUTE BC, GC, Tokyo, Japan). A craniotomy (diameter 1.0∼1.5 mm) for imaging, centered at the stereotaxic coordinate of anterior-posterior ∼−2.5 mm and medial-lateral ∼3.0 mm from the bregma, was made using a dental drill above the somatosensory cortex. The dura mater was surgically removed. Care was taken to avoid any damage to the pial vessel and cortical surface. Another small craniotomy (diameter 0.5 mm) was made approximately 2.0 mm posterior to the imaging window for intracranial electroencephalogram (EEG) so that the electrode will not make a physical intrusion to the imaged area, while measuring the population activity in the vicinity of the imaged area. The craniotomies were perfused with warmed Hepes-Ringer solution (∼35°C) to maintain surface temperature of the brain during the experiment. All experimental protocols were approved by the RIKEN Institutional Animal Care and Use Committee.

### Dye loading and in vivo imaging

Multi-cell bolus loading was performed to load neurons and glial cells with Ca^2+^ sensitive fluorescence indicator Oregon Green 488 BAPTA-1 (OGB-1) and astrocyte specific fluorescence marker Sulforhodamine 101 (SR101) [20], [21]. Briefly, the dye containing solution was prepared with the following reagents: Oregon Green 488 BAPTA-1 AM (50 µg, O-6807, Molecular Probes-Invitrogen, CA, USA), Pluronic F-127 (5 µl, P3000MP, Molecular Probes-Invitrogen, CA, USA), phosphate buffered saline (42 µl, pH 7.4, PBS), SR101 (3 µl of 1 mM solution, S-359, Molecular Probes-Invitrogen, CA, USA). The dye containing solution was filtered with 0.22 µm pore Millex -GV (Millipore, MA, USA). A quartz glass micropipette of tip diameter approximately 1∼2 µm, containing 5 µl of the dye mixture was slowly inserted into the cortex (200 µm below the pia) using an electronic fine manipulator (EMM-3, Narishige, Tokyo, Japan). The dye mixture was introduced to the tissue by air pressure application to the pipette (70 kPa, 1 min). The craniotomy was then covered with agarose (1.5% w/v in PBS) and sealed by placing a thin glass coverslip (3 mm×3 mm, thickness<0.12 mm, Matsunami Glass Ind., Osaka, Japan). The cranial window was secured by dental cement. Imaging was performed with a custom-modified Olympus FV-1000 based two-photon microscope. Chameleon XR laser (Coherent Inc. Santa Clara, CA, USA) was used as the excitation light source. The group velocity dispersion was compensated by a prism coupled pre-chirper and the beam diameter was adjusted by a Kepler telescope. The output power of the laser power was adjusted by a motorized graded neutral density filter or polarizing prism. Laser power (mW) measured after the objective lens (LUMPLANFL40XW, N.A. 0.8, Olympus, Tokyo, Japan) was 32.8±9.0 and 65.5±11.1 for L1 and L2/3, respectively with laser irradiation time of 2 µs/pixel. Fluorescence from OGB-1 and SR101 were simultaneously monitored by applying appropriate dichroic mirrors and filters. Fluorescent dye loaded areas (320 µm×320 µm) were imaged for thirty minutes at 0.5 Hz with 320×320 pixels from 38 urethane anesthetized rats. For pharmacological experiments, inhibitors (pyridoxal-phosphate-6-azophenyl-2′,4′-disulfonic acid (PPADS: 1 mM), atropine (1 mM), tetrodotoxin (TTX: 2 µM)) were applied on the cranial window at least 30 min before imaging [46], [47]. Systemic injection of 2-methyl-6-(phenylethynyl)-pyridine (MPEP) was performed 30 min before imaging [16]. MPEP and PPADS were purchased from Tocris Bioscience (Bristol, UK). Atropine sulfate and TTX were purchased from Sigma-Aldrich (MO, USA).

### Electrophysiology

A quartz glass pipette (resistance 2∼3 MΩ) containing physiological saline was inserted 300∼400 µm below the pial surface with an insertion angle of 30 degree. EEG was measured by taking a differential signal between the microelectrode and a reference signal at the neck of the animal. The EEG signal was amplified by 1,000 fold and bandpass filtered between 0.1 and 4k Hz (Multiclamp 700B, Molecular Devices, CA, USA). Electrocardiogram (EKG) was monitored continuously using metal electrodes by taking a differential signal between chest electrode and reference electrode located at a foot of the subject. EEG, EKG, and a synchronizing signal from the two-photon microscope were simultaneously digitized at 25 kHz using a commercial data acquisition card (M-series PCI-6259 National Instruments, TX, USA). The data acquisition software was written with LabView (National Instruments, TX, USA).

### Immunohistochemistry

After the imaging measurements were made, the subjects were transcardially perfused with physiological saline, followed by 100 ml of 0.1 M phosphate buffer containing 4% paraformaldehyde. Coronal sections corresponding to the imaged area were cut at 60 µm thickness in 0.1 M phosphate buffer (pH 7.4) using a vibratome (Pro-7 Linear Slicer, Dosaka EM, Kyoto, Japan). For the detection of cytoarchitectural difference of cortical layers (Figure 1C), slices were immunostained using primary antibodies of anti-S100β rabbit polyclonal antibody (1∶1000 dilution, Product ID 37, Swant, Switzerland) and anti-NeuN mouse monoclonal antibody (1∶1000 dilution, Product ID MAB377, Chemicon-Millipore, MA, USA). For the assessment of craniotomy condition (Figure 4C–D), slices were immunostained using anti-glial fibrillary acidic protein (GFAP) mouse monoclonal antibody (1∶1000 dilution, Product ID G3893, Sigma-Aldrich, MO, USA) and anti-ionized calcium-binding adaptor molecule 1 (Iba-1) rabbit polyclonal antibody (1∶1000 dilution, Product ID 019-19741, Wako Pure Chemicals, Osaka, Japan). Subsequently, slices were incubated with fluorescent secondary antibodies of Alexa Fluor 488 conjugated anti-mouse antibody (1∶2000 dilution, Product ID A11001, Molecular Probes-Invitrogen, CA, USA) and Cy3 conjugated anti-rabbit antibody (1∶2000 dilution, Product ID 711-166-152, Jackson Immunoresearch Laboratories, PA, USA ). Fluorescence images were obtained with Olympus BX-51 fluorescence microscope (Figure 1C) or Olympus FV-1000 confocal microscope (Figure 4C–D). Identical laser power was used for scanning the craniotomy and the corresponding contralateral control regions.

### Data analysis

Fluorescence signal was quantified by measuring the mean pixel value of a manually selected somatic area for each frame of the image stack using ImageJ software (http://rsb.info.nih.gov/ij/). The values were exported to MATLAB (Mathworks, MA) and the fluorescence change was computed. Ca^2+^ surges of astrocyte were detected when fluorescence intensity of OGB-1 reached higher than 117% of baseline fluorescence intensity. Baseline fluorescence intensity was defined as the mean of the lower two-third of the fluorescence intensity histogram for each astrocyte during the thirty minute imaging. Duration of each Ca^2+^ surge was defined as duration of fluorescence intensity above 50% of its peak intensity during a Ca^2+^ surge. Pairwise crosscorrelograms between pairs of astrocytes were computed by standardizing the fluorescence signal to be the Z score, so that the height of the crosscorrelogram represents the Pearson correlation coefficient. Synchrony of Ca^2+^ traces of a pair of astrocytes was calculated using the Fisher's exact test in which traces of fluorescence intensity of Ca^2+^ indicator was divided into two periods, i.e. periods with or without Ca^2+^ surge, for each imaging frame. For astrocytic process analyses, the Pearson correlation coefficients were computed to assess the synchrony of Ca^2+^ dynamics. All data are expressed as mean±standard deviation (SD), except where otherwise noted. The Student's t-test, Wilcoxon signed-rank test (for two groups) and ANOVA, followed by the Tukey multiple comparison test (for three or more groups) were used to determine the statistical significance (p<0.05) of differences. Statistical analyses were performed by programs written with MATLAB.

## Supporting Information

Figure S1 Representative Time Course of Spontaneous Ca^2+^ Surges of Astrocytes in Layer 2/3. A, Representative *in vivo* image of layer 2/3. Each trace show normalized fluorescence intensity of Ca^2+^ indicator OGB-1 from numbered astrocytes (1–6) in (A). Small red dots indicate period of Ca^2+^ surge. Vertical position of each trace was adjusted arbitrary to improve visibility. Scale bar: A, 100 µm; B, 50%.
(1.90 MB TIF)

Figure S2 A, Amplitude of EEG was increased by MPEP. (A1) Representative EEG traces of somatosensory cortex before and after MPEP injection. Rats were anesthetized with urethane of dosage:1.2 g/kg) to induce desynchronized state of EEG (Binns and Salt, 2001). (A2) Comparison of power spectrum densities before (black) and after (red) MPEP injection. Traces represent mean±SEM (n = 5). MPEP significantly increased the power of slow wave (0.5–2 Hz) (control, 32.6±1.9; MPEP, 44.8±1.2, t-test, ***p<0.001). B, ATP-induced Ca^2+^ surge was suppressed by PPADS. (B1) Average traces±SEM (n = 8) of astrocytic Ca^2+^ responses to ATP in the presence (red) or absence (black) of PPADS. ATP (100 mM) was pressure-injected into the somatosensory cortex using a pipette (tip ∼1 µm, 70 kPa, 1 sec). (B2) Comparison of peak amplitude of ATP-induced Ca^2+^ surges of astrocytes. PPADS significantly reduced the Ca^2+^ responses (control, 122±4%; PPADS, 110±3%, n = 8, t-test, ***p<0.001). C, Atropine prevented EEG desynchrony induced upon a stimulation of the nucleus basalis (NB). (C1) Representative EEG traces of somatosensory cortex with NB stimulation in the absence (upper trace) or presence (lower trace) of atropine. Immediate after NB stimulation, slow wave transiently disappears for a time period of three to five seconds (single arrow), while slow wave persists after NB stimulation in the presence of atropine (asterisk). NB was stimulated with a bipolar tungsten electrode (200 µA, 100 Hz, 50 pulses, pulse duration 500 µs). (C2) The effectiveness of NB stimulation was quantified by measuring ratio of EEG amplitude 1∼3 sec prior to and after NB stimulation. Values larger than 1 indicate decrease in EEG amplitude by NB stimulation. Atropine significantly reduced effectiveness of NB stimulation (control, 1.9±0.3; atropine, 1.2±0.5, n = 10, t-test, ***p<0.001).
(1.02 MB TIF)

Figure S3 Representative Traces of Ca^2+^ Activities of Processes of an Astrocyte in Layer 2/3. A, Soma and processes of an astrocyte in layer 2/3 were identified with astrocyte specific SR101 image. ROIs were selected in the same manner as Fig. 9A in three primary processes (proc) from an astrocyte with the proximal (p) and the distal (d) parts. As a control, one ROI was placed at neuropil at least 100 µm away from the astrocyte. B, Time course of the Ca^2+^ indicator (OGB-1) signal is plotted for each ROI within a single astrocyte in L2/3. ROIs were selected in the same manner as Fig. 9A in three primary processes (proc) from an astrocyte with the proximal (p) and the distal (d) parts. As a control, one ROI was placed at neuropil at least 100 µm away from the astrocyte. Scale bar: A, 20 µm; B, 100%.
(1.77 MB TIF)

Video S1 Imaging of Spontaneous OGB-1 Fluorescence Dynamics in Cortical Layer 1 In Vivo. The movie was taken in the layer 1 (47 µm below the pia) of the primary somatosensory cortex loaded with the Ca2+ sensitive fluorescence dye OGB-1 (green) and the astrocyte specific marker SR101 (red). The movie represents the same area as figure 1 A (Layer 1). The movie was compressed sixty times to display 30 min of the experiments in 30 s. Spontaneous increase of fluorescence intensity of the Ca2+ dye is observable in astrocytes. The avi-movie file was compressed with codec “IndeoR video 5.10” for reviewing process.
(8.73 MB MOV)

Video S2 Imaging of Spontaneous OGB-1 Fluorescence Dynamics in Cortical Layer 2/3 In Vivo. The movie was taken in the layer 2/3 (192 µm below the pia) of the primary somatosensory cortex stained with OGB-1 (green) and SR101 (red) for thirty minutes. The movie represents the same area as figure 1 A (Layer 2/3). The time compression rate is identical to Video S1. Spontaneous increases of intensity of the Ca2+ dye are observable in astrocytes and neurons. The avi-movie file was compressed with codec “IndeoR video 5.10” for reviewing process.
(9.97 MB MOV)
