# Supplementary figures and images for: Cortical Layer 1 and Layer 2/3 Astrocytes Exhibit Distinct Calcium Dynamics In Vivo
Source: PLoS One. 2008 Jun 25;3(6):e2525. doi: 10.1371/journal.pone.0002525 (PMC2424136; doi:10.1371/journal.pone.0002525)

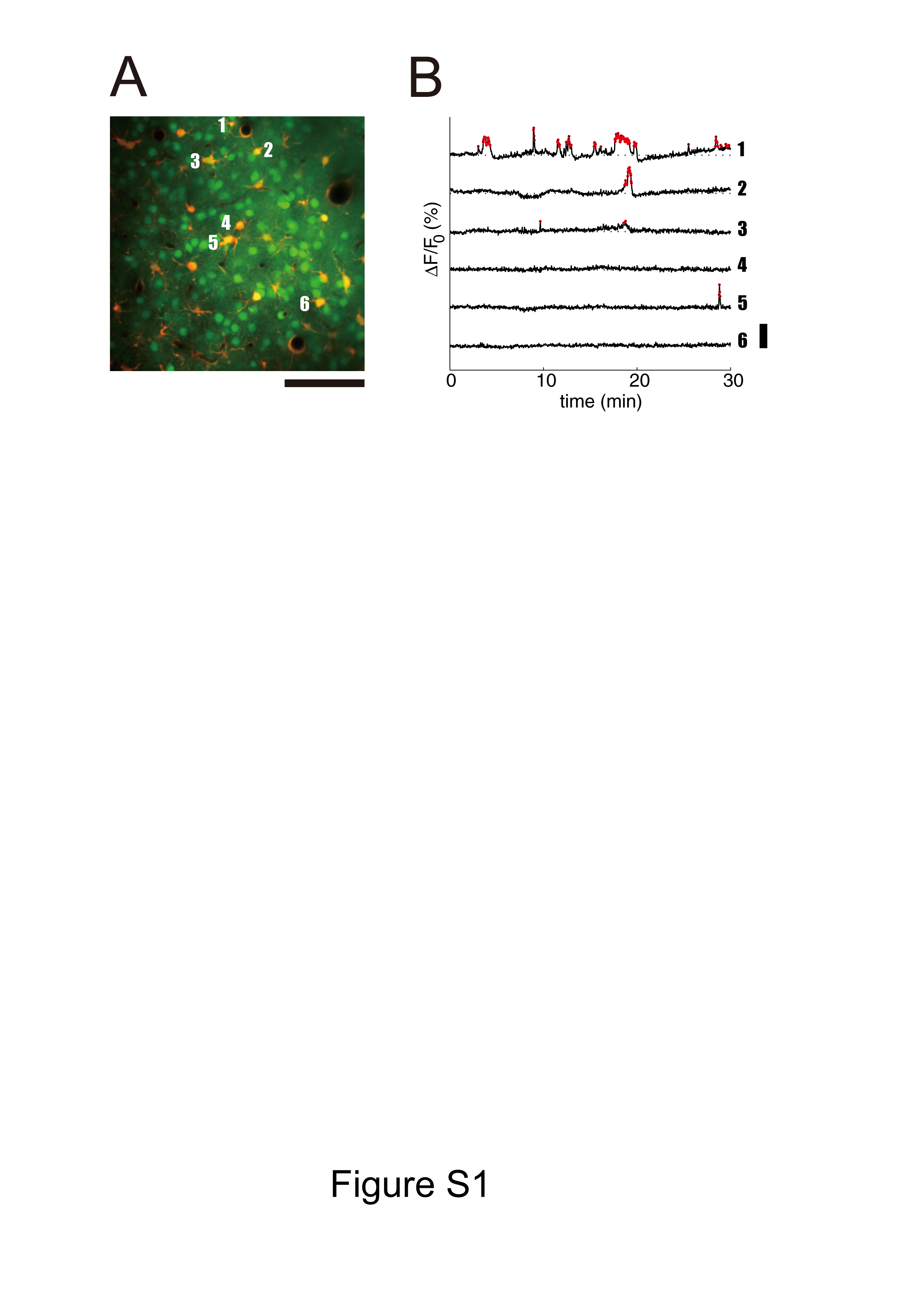

Supplement: Figure S1 — Representative Time Course of Spontaneous Ca2+ Surges of Astrocytes in Layer 2/3. A, Representative in vivo image of layer 2/3. Each trace show normalized fluorescence intensity of Ca2+ indicator OGB-1 from numbered astrocytes (1–6) in (A). Small red dots indicate period of Ca2+ surge. Vertical position of each trace was adjusted arbitrary to improve visibility. Scale bar: A, 100 µm; B, 50%. (1.90 MB TIF) [file pone.0002525.s001.tif]

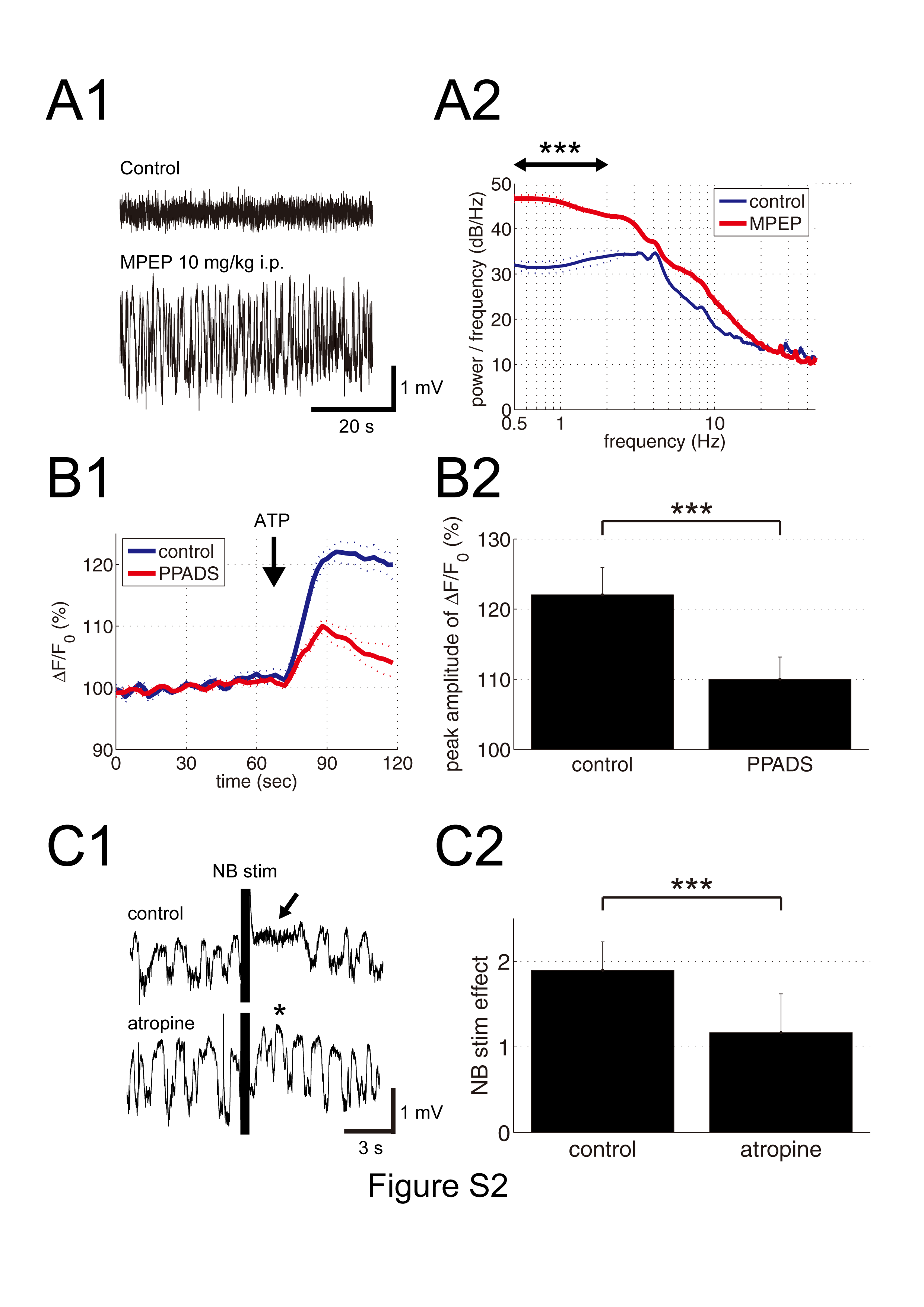

Supplement: Figure S2 — A, Amplitude of EEG was increased by MPEP. (A1) Representative EEG traces of somatosensory cortex before and after MPEP injection. Rats were anesthetized with urethane of dosage:1.2 g/kg) to induce desynchronized state of EEG (Binns and Salt, 2001). (A2) Comparison of power spectrum densities before (black) and after (red) MPEP injection. Traces represent mean±SEM (n = 5). MPEP significantly increased the power of slow wave (0.5–2 Hz) (control, 32.6±1.9; MPEP, 44.8±1.2, t-test, ***p<0.001). B, ATP-induced Ca2+ surge was suppressed by PPADS. (B1) Average traces±SEM (n = 8) of astrocytic Ca2+ responses to ATP in the presence (red) or absence (black) of PPADS. ATP (100 mM) was pressure-injected into the somatosensory cortex using a pipette (tip ∼1 µm, 70 kPa, 1 sec). (B2) Comparison of peak amplitude of ATP-induced Ca2+ surges of astrocytes. PPADS significantly reduced the Ca2+ responses (control, 122±4%; PPADS, 110±3%, n = 8, t-test, ***p<0.001). C, Atropine prevented EEG desynchrony induced upon a stimulation of the nucleus basalis (NB). (C1) Representative EEG traces of somatosensory cortex with NB stimulation in the absence (upper trace) or presence (lower trace) of atropine. Immediate after NB stimulation, slow wave transiently disappears for a time period of three to five seconds (single arrow), while slow wave persists after NB stimulation in the presence of atropine (asterisk). NB was stimulated with a bipolar tungsten electrode (200 µA, 100 Hz, 50 pulses, pulse duration 500 µs). (C2) The effectiveness of NB stimulation was quantified by measuring ratio of EEG amplitude 1∼3 sec prior to and after NB stimulation. Values larger than 1 indicate decrease in EEG amplitude by NB stimulation. Atropine significantly reduced effectiveness of NB stimulation (control, 1.9±0.3; atropine, 1.2±0.5, n = 10, t-test, ***p<0.001). (1.02 MB TIF) [file pone.0002525.s002.tif]

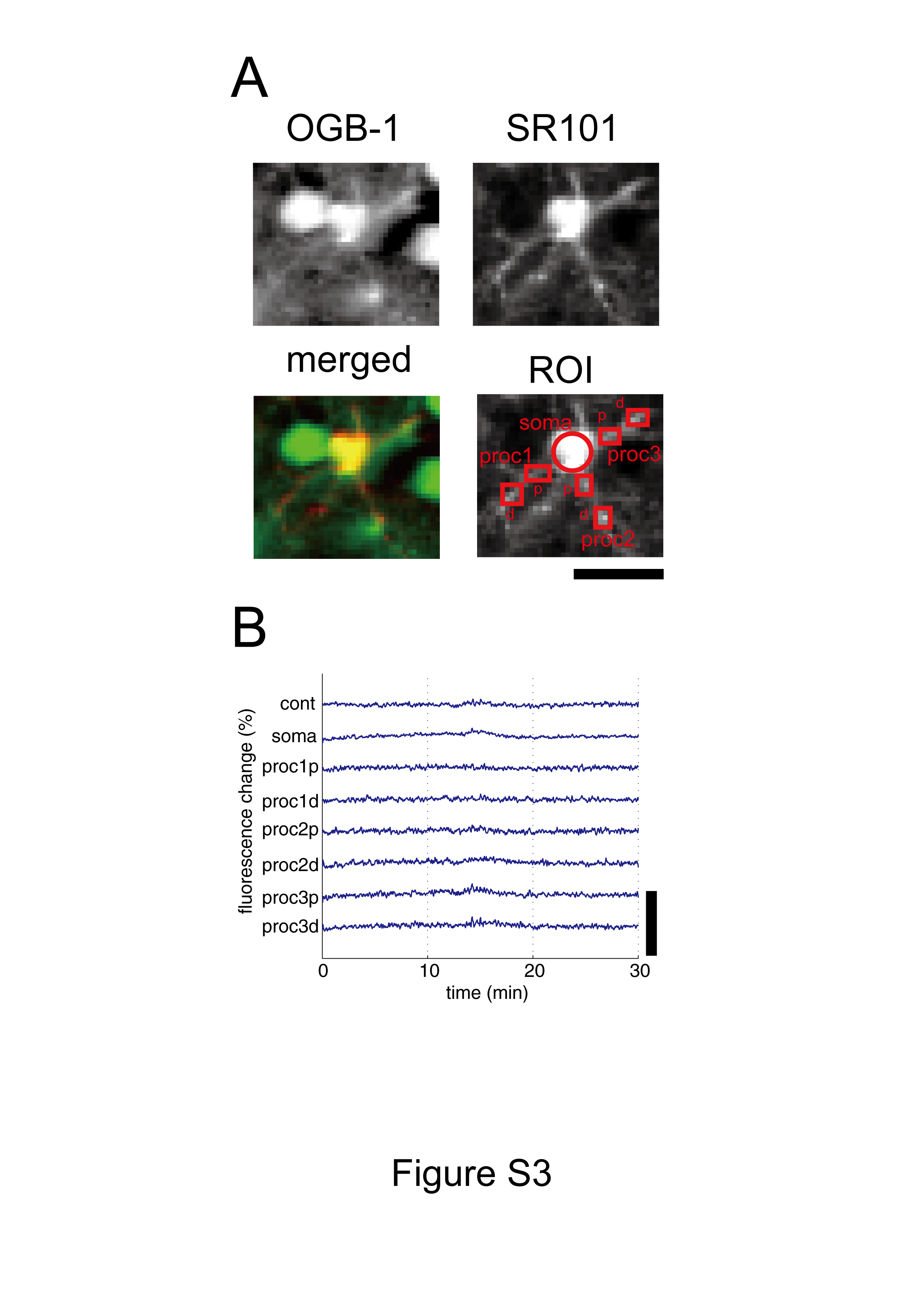

Supplement: Figure S3 — Representative Traces of Ca2+ Activities of Processes of an Astrocyte in Layer 2/3. A, Soma and processes of an astrocyte in layer 2/3 were identified with astrocyte specific SR101 image. ROIs were selected in the same manner as Fig. 9A in three primary processes (proc) from an astrocyte with the proximal (p) and the distal (d) parts. As a control, one ROI was placed at neuropil at least 100 µm away from the astrocyte. B, Time course of the Ca2+ indicator (OGB-1) signal is plotted for each ROI within a single astrocyte in L2/3. ROIs were selected in the same manner as Fig. 9A in three primary processes (proc) from an astrocyte with the proximal (p) and the distal (d) parts. As a control, one ROI was placed at neuropil at least 100 µm away from the astrocyte. Scale bar: A, 20 µm; B, 100%. (1.77 MB TIF) [file pone.0002525.s003.tif]
